# From development to implementation through a RE-AIM lense: lessons learned from a qualitative process evaluation of workplace health promotion workshops in long-term care

**DOI:** 10.3389/fpubh.2026.1917030

**Published:** 2026-09-04

**Authors:** Judith Czakert, Fabian Voigt, Anny Warncke, Sandra Jankow, Julia Berschick, Nina Effenberg, Anne Schirmaier, Georg Seifert, Christian Kessler, Wiebke Stritter

**Affiliations:** 1 Charité Competence Center for Traditional and Integrative Medicin (CCCTIM), Charité - University Medical Center Berlin, Corporate Member of Freie Universität Berlin and Humboldt-Universität zu Berlin, Berlin, Germany; 2 Department of Integrative Health Care and Prevention, University of Augsburg & University Hospital Augsburg, Augsburg, Germany; 3 Department of Internal and Nature-Based Therapies, Immanuel Hospital Berlin, Berlin, Germany

**Keywords:** health services research, long-term care, occupational health, process evaluation, qualitative research, re-aim, staff wellbeing, workplace health promotion

## Abstract

**Background:**

Workplace Health Promotion (WHP) in long-term care facilities is essential to support staff wellbeing and sustain workforce capacity, yet implementing such interventions remains challenging. Within the pilot project “*Healthy Care Cares for Health”*, WHP workshops were developed and implemented. To strengthen the feasibility of future WHP projects in long-term care facilities, this study pursued two objectives: first, to present the development and implementation of the workshops to ensure traceability and enable adaptation in future setting-specific WHP projects; second, to derive implications for practice, guided by the Reach, Effectiveness, Adoption, Implementation, and Maintenance (RE-AIM) framework, based on the barriers and facilitators encountered during implementation.

**Methods:**

Three WHP workshops were developed in an iterative multi-step procedure. Implementation in one pilot facility was evaluated through a qualitative process evaluation. Data collection included (1) systematic documentation of the development workflow and (2) structured observations during the workshops. Data were analyzed through qualitative content analysis with a deductive approach, complemented by inductive coding. To structure and clarify the lessons learned derived from the findings, the RE-AIM framework was applied.

**Results:**

The development of the workshops comprised seven phases: expert selection, onboarding, individual and collaborative development, first implementation, adaptation, and second implementation. Findings from the process evaluation showed that levels of engagement varied. While approximately one-third of participants were actively involved, others remained passive or even showed signs of resistance. Key challenges included motivation, perceived content relevance, group dynamics, fatigue, and language barriers. The adjustments that were made, based on observations and feedback from the first workshop round, led to improved structure, delivery, and acceptance of the workshops. The evaluation revealed two key areas for improvement: (1) organizational preparation; (2) workshop design, including content, structure, and methodology. Participation proved to be a central factor for motivation, serving both as a prerequisite for successful WHP initiatives and an indicator of successful implementation. Using the domains of the RE-AIM framework, the lessons learned were summarized and synthesized into implications for future WHP interventions.

**Conclusions:**

This study sheds light on the development and implementation of WHP interventions in long-term care settings. Active participation and organizational readiness were identified as crucial factors for successful implementation. The key lessons learned were structured according to the RE-AIM framework to derive practical implications, enabling a transferable synthesis of the results. These offer practical guidance on designing, adapting, and implementing WHP interventions in long-term care contexts.

**Clinical trial number:**

The pilot project HCCH has been registered with the German Clinical Trials Register (DRKS) under the registration number DRKS00036359 (date of registration: 17 March 2025).

## 1 Introduction—“healthy care cares for health”

Working conditions of nurses are typically characterized by specific determinants of health (e.g., irregular sleeping patterns [e.g., (1)], physical hazards (e.g., high physical workload, exposure to hazardous substances [e.g., (2)]), and mental challenges (e.g., high stress levels, time and staff shortages, violence experiences [e.g., (3)]). The resulting challenges for body and mind are associated with increased health risks, including cardiovascular diseases, metabolic disorders, low back pain, depression, and burnout (4–6). These health risks may also have economic consequences, as reflected by the increasing number of sickness-related absences and rising tendency toward early retirement among employees in Germany's healthcare sector (7, 8). Nursing staff in long-term care facilities are particularly affected by the work-related health challenges and the resulting economic risks (7, 9, 10).

The background outlined calls for workplace health promotion (WHP) programs for nurses in general and long-term care facility staff in particular (9, 11). The positive effects of WHP programs on physical and mental health (12, 13), on sickness-related absenteeism, and long-term workability of nurses, are already well known (14). However, the sustainable implementation of such programs is characterized by challenges (15). Previous studies (16, 17) have shown that barriers for the long-term success of WHP programs for nurses are considered to be instability in working conditions, stress, and limited time due to staff shortages. Accordingly, one crucial aspect is the implementation of WHP initiatives during working hours. WHP measures are often perceived as an additional burden by the target group, which in turn can have a negative effect on motivation to engage with the interventions. Equally, factors such as a combination of management-supported structural prevention and low-threshold behavioral prevention measures are identified as necessary preconditions and facilitators for successful WHP programs for nurses (ibid.).

### 1.1 Pilot study “healthy care cares for health”

The integration of perspectives and approaches from Traditional, Complementary, and Integrative Medicine (TCIM) into WHP programs may contribute to enhancing individual wellbeing, motivation, and long-term engagement of the target group: TCIM offers a largely untapped potential with its holisticly and resource-oriented perspectives, a view that is reinforced by the World Health Organization's (WHO) policy- and advocacy-related work in favor of its integration into WHP (18). Practices such as mindfulness exercises, breathing techniques, yoga, aromatherapy, and healthy plant-based nutrition, show promising effects in terms of stress reduction, enhanced physical wellbeing, and improved self-regulation capacities [ex.: (19–23)]. Not only has evidence from a recent study indicated that the level of acceptance of such approaches is high among care staff in long-term care settings (24). They have also shown to yield positive outcomes for staff health, wellbeing, and the quality of nurse-patient relationships (25, 26). Based on recent data on the use of TCIM measures in Germany (27), it can be assumed that a substantial proportion of nurses are already familiar with one or more TCIM practices and demonstrating a generally positive attitude toward them. This openness and motivation, particularly when combined with opportunities for participatory involvement in their development and implementation, can be expected to enhance the general engagement with WHP programs. To date, to the knowledge of the authors, there are no WHP programs that combine explicitly TCIM approaches with multi-modality and participation, specifically tailored to (nursing) staff in long-term care facilities (28).

To explore and use this potential for WHP interventions, the pilot project “*Healthy Care Cares for Health” (HCCH)* aims to develop, implement, and evaluate a WHP program for long-term care facilities in Germany that includes TCIM strategies. Further characteristics of *HCCH* are the target group participation in the program's development and implementation at different levels, and a multimodular structure of the program. The multimodular structure allows the program to adapt to the specific needs and conditions of various long-term care facilities and their staff members, addressing the call for those tailored WHP interventions that are specific to the target group and setting (11). The continuous involvement of the target group in the pilot project at various levels of participation (29) is intended to encourage a sense of ownership, create a multiplier effect, and thus build a strong foundation for the program's long-term sustainability.

#### 1.1.1 “Healthy care cares for health”—context, setting, intervention

The pilot project is scheduled to run for 5 years, starting in September 2022 with the conduction of a scoping review (28). The scoping review compiled and synthesized both existing scientific evidence and the practical experiences of health-care professionals in Germany. The choice of a long-term care facility was informed by preconditions identified through the review findings. The selected pilot facility is located in Berlin, Germany. When the facility was enrolled in the study (January 2024), the staff comprised a total of 33 employees, including a mix of professional groups (nursing staff, residential care assistants, management/ administration specialists, facility management, social worker). The facility did then accommodate and continues to do so 53 residents with varying levels of care dependency. It is affiliated with a religious welfare association that operates two additional long-term care facilities in Berlin, Germany.

In order to explore the health-related opportunities and challenges of the pilot facility, its work processes, and staff, a needs assessment was carried out in July and August 2024 (24). The results provide a foundation for the further development of the WHP program, enabling the program's adaptation to the specific characteristics of the target group and setting. It is particularly noteworthy that some staff members already regularly use approaches associated with TCIM for themselves, such as mindfulness exercises, breathing exercises, and deliberate engagement with nature and art. This may be interpreted as an indication of interest, motivation, and openness toward a TCIM-based WHP program (ibid.).

#### 1.1.2 “Healthy care cares for health”—two-phase WHP intervention

The WHP program is planned as a multi-modular intervention that includes TCIM principles. Its multi-modular character is reflected in a two-phase implementation structure:

In the **initial phase**, three thematic workshops are conducted, focusing respectively on nutrition, physical activity, and stress regulation. Four distinct *Health Promotion Bundles (HPB)* were developed for each workshop. Each *HPB* addresses a specific focus within its respective topic area and comprises 10 to 20 micro-level exercises targeting physical and mental wellbeing. At the end of each workshop, participants select one *HPB* to be implemented in the pilot facility, allowing them to participate in shaping the intervention's direction.

In the **second phase**, the three selected *HPBs* will be implemented in the pilot facility. This process involves volunteer staff members (health mentors) who participate in regularly scheduled health circles. Health circles are structured, recurring meetings established to plan and coordinate the implementation of the *HPB*. The health circles are intended to serve two objectives: First, to enable a setting-specific implementation of the *HPB* that, with the explicit involvement of selected health mentors, accounts for the setting's parameters as well as participants' needs and preferences. Second, to establish a recurring meeting format which staff can further develop in order to sustain the intervention in the long term. Ultimately, the health circle is intended to become an organizationally embedded, lasting structure within the pilot facility. The aim of this second phase is to support sustainable, long-term health promotion. Implementation of the health circles is scheduled between January and August 2026. The implementation of the *HPB* will start in September 2026.

This paper presents and discusses the **initial phase** of the project: the development, implementation and evaluation of three health promotion workshops. To organize the evaluation's core findings in a systematic way, while considering the multi-dimensional aspects of complex interventions, the Reach, Effectiveness, Adoption, Implementation, and Maintenance (RE-AIM) framework is used.

### 1.2 The RE-AIM framework as a structured approach to synthesizing key learnings from implementation research

The RE-AIM framework is a widely applied model for evaluating and informing the translation of health interventions into real-world settings (30). By encompassing five dimensions relevant for implementation research (Reach, Effectiveness, Adoption, Implementation, and Maintenance), it enables a systematic assessment of an interventions' impact, including implementation-related factors that influence delivery and outcomes [ibid.]. Originally developed to enhance the external validity of health promotion interventions (31), RE-AIM has evolved into a framework that is also used to guide planning, evaluation, and reporting, as well as to structure findings from implementation studies (11). RE-AIM provides an analytic structure to organize and synthesize key learnings from the evaluation of complex WHP interventions systematically by capturing multiple dimensions in a transparent way (30). Its multi-dimensional perspective supports the identification and categorization of factors related to feasibility, adoption, and practical implementation, as well as long-term sustainability. Thus, applying RE-AIM to organize and synthesize key findings from implementation research provides a structured approach to present insights into the feasibility and practical applicability of complex, multifaceted health interventions, such as workplace health promotion (WHP) interventions in long-term care facilities.

### 1.3 Research objectives and question

To strengthen the feasibility of future WHP projects in long-term care facilities, this paper pursues two objectives: First, presentation of the development and implementation of the workshops to ensure traceability and allow for adaptation in future projects. Second, derivation of RE-AIM-guided practice implications based on the pitfalls and facilitators encountered during the implementation of the workshops.

The underlying research question is: What lessons can be drawn from the development and implementation of the workshops to inform future workplace health promotion (WHP) projects in long-term care facilities?

## 2 Methods—intervention development and evaluation

To clarify context, transparency, and to enhance adaptability of this study, the workshop development is described first, followed by an overview of the process evaluation design with regards to sampling, data collection and analysis.

### 2.1 Workshop development

Part one of the WHP program consists of three workshops, designed to start a sustainable, ongoing health promotion process with the pilot facility's entire staff (nursing staff, residential care assistants, management/ administration specialists, facility management, social worker), focused on, but not limited to, nurses. The topics of the workshops (1. nutrition, 2. stress regulation, and 3. physical activity) are aligned with the key areas of action for WHP, defined in the official German guidelines on prevention (14). The workshops were developed in collaboration with selected experts and practitioners (see chapter 3.1.) to ensure contextual relevance and feasibility. The following principles and conditions for the development of the workshops were established in advance:

#### 2.1.1 Workshops: underlying principles and conditions

A fundamental requirement for developing the workshops was that all workshops must be in accordance with all predefined program specifics, such as including TCIM-based content, combined with healthy physical exercises, active involvement of the participants, and adjustable to the setting and the working conditions. To stimulate participants' motivation to engage in WHP programs, some guiding principles were defined prior to the development of the workshops. These principles structured the sessions in a recurring pattern, combining three elements: the transfer of information to provide a shared basis of knowledge, self-awareness exercises to foster reflection and deepen understanding, and interactive exchanges to encourage dialogue and experience sharing.

Another predefined feature of all workshops concerned the nature of the exercises. To ensure feasibility within the demanding routines of long-term care facilities, micro-exercises were selected. A key advantage of these micro-exercises lies in their minimal time requirements and their potential for seamless integration into daily work routines, thereby enhancing both feasibility and acceptance among staff (32, 33).

#### 2.1.2 Workshops: content and evidence-base

The content of the workshops is based on the following background.

##### 2.1.2.1 Workshop 1 (nutrition)—“boost your health: eat the natural way”

Nursing staff predominantly work in shifts, which is associated with increased health risks. These include a high body mass index (BMI) and an increased prevalence of overweight and obesity (34, 35). Diet is a major contributing factor: shift working is associated with unhealthier meal timing and skipping meals and poorer diet quality (34, 36). These dietary and lifestyle factors, in turn, may increase the likelihood of developing cardiometabolic diseases such as hypertension, type 2 diabetes, and coronary heart disease, which are recognized as leading causes of premature mortality worldwide (37). There is awareness that a balanced diet can promote wellbeing and reduce the burden of diet-related diseases (38), support physical and mental performance and have a beneficial impact on perceived stress (39, 40). Furthermore, nutrients are required for a strong immune system as well as for physical and mental fitness—factors that can enhance to cope with working life's challenges (41, 42). Work-related conditions, such as shift work, time pressure, and irregular breaks make it difficult for nurses to implement a healthy diet that is both enjoyable and tasty. To counteract, shift workers need adjustable low-threshold strategies to implement healthy eating habits even in stressful work environments such as long-term care facilities.

##### 2.1.2.2 Workshop 2 (stress regulation)—“boost your health: relax the natural way”

Absenteeism due to mental illness has been steadily rising in nurses' workplace environments (3). Sickness absence rates in nursing exceed the cross-industry average, with staff in long-term care facilities most affected (43). Nursing work is associated with numerous psychological stressors; these stressors can result in reduced performance, job dissatisfaction, sleep and concentration disturbances, emotional exhaustion, burnout, depression, musculoskeletal disorders, and health-compromising behaviors (e.g., smoking, physical inactivity, unhealthy diet, alcohol or medication use) (44). Consequently, the profession's attractiveness declines, contributing to a growing shortage of qualified staff, a trend further exacerbated by demographic changes (45).

Addressing these challenges requires multifaceted WHP strategies to enhance both the attractiveness and health-promoting nature of the staff's work. WHP measures should focus on work conditions (e.g., management culture, risk prevention) and encourage healthy behaviors among employees, including physical activity, relaxation, and nutrition (46). Stress management interventions represent an effective component of such behavioral prevention. Mindfulness exercises positively influence physiological parameters, such as blood pressure, heart rate, and pain, as well as psychological outcomes, including stress and anxiety, and are associated with higher job satisfaction, empathy, and work engagement (47). Brief mindfulness breaks, deep-breathing exercises, and short practices, such as 5-min meditations or hand massages, can be seamlessly integrated into daily routines and reduce stress effectively (48–50). Essential-oil based interventions, such as aromatherapy, may further alleviate stress and anxiety (51).

Social stressors, including bullying and discrimination, also significantly affect nursing staff. Accordingly, workplace health promotion programs should address communication processes to enhance teamwork and overall job satisfaction (52).

##### 2.1.2.3 Workshop 3 (physical activity)—“boost your health: move the natural way”

Research has demonstrated that physical exercise plays a pivotal role in promoting health and wellbeing in the workplace. The benefits of physical exercise on health and wellbeing in the workplace are widely recognized (53–55). This is especially relevant in the nursing profession in different settings as employees are confronted with a variety of occupational risks, both physical and mental, that can affect their wellbeing and job performance (56–58). Organizational factors such as shift work, long working hours, and staff shortages are known to influence physical activity as well as sedentary behavior increasing the risk of health problems (56). Additionally, the physical demands of nursing, such as lifting heavy weights, frequent carrying, and working in stressful postures, can contribute to musculoskeletal disorders. Targeted physical exercise has been shown to effectively reduce these work-related issues and reduce stress levels (59–61). Exercise in the workplace not only promotes physical health but also strengthens social relationships (62–64), which can have a positive impact on mental wellbeing. Frequent and active short breaks from sedentary behavior were pointed out to effectively improve mood in daily life (65). Study results show that even brief exercise breaks during working hours can furthermore boost nurses' productivity and efficiency, reduce absenteeism, and enrich workplace relationships (66, 67). Moreover, it is noteworthy that incorporating physical activity into the workday can positively impact individuals' exercise habits outside of work (68).

### 2.2 Workshop evaluation

The research design was guided by an exploratory perspective, aiming to capture the intervention as it unfolded in practice and to provide a descriptive snapshot of the intervention's state and process (69). To generate in-depth, contextual insights into the development and implementation of the intervention and explore its feasibility in the long-term care pilot facility, a qualitative, process evaluation (70) based on observational data was conducted. The observational approach was chosen because it provides a comprehensive understanding of processes, interactions, and contextual factors in real-life settings that are difficult to access through self-report methods, such as interviews or questionnaires (71, 72).

The study was conducted by researchers trained in qualitative research and with a background in nursing (research). This combination of methodological expertise and firsthand experience in nursing allowed for an approach that was both open and sensitive to the contextual conditions and specific characteristics of the target group and setting. To ensure methodological rigor, the findings were regularly reviewed and discussed with colleagues trained in qualitative research.

#### 2.2.1 Sampling, data collection, and data analysis

The **sampling** strategy was informed by the data collection method, which relied on qualitative observations conducted during the workshops. In this context, the sampling emerged alongside data collection: all workshop participants – potentially encompassing all employees of the pilot facility – were included in the observations. All staff members (*n* = 33 at the time of the first workshop in March 2025), rather than nursing staff alone, were eligible to participate, in line with the HCCH project's aim of promoting workplace health across the entire long-term care facility setting rather than within a single professional group. Thus, the sample can be described as a convenience sample. All participants provided informed consent prior to data collection. The characteristics of the participants are derived from two sources: (a) the pilot facility's basic personnel data and (b) the sociodemographic data from a baseline survey conducted in January 2025 as part of the planned PROMS-based effectiveness evaluation of the *HCCH* project. More than half of the staff members were nursing assistants (54.5 %), alongside residential care assistants for social support (12.1 %), registered nurses (18.2 %), administrative staff (9.1 %), and housekeeping staff (6.1 %). Of the 33 staff members, 29 took part in the baseline survey in January. The majority of these were female (79.3 %) and middle-aged: 27.6 % of the participants were younger than 40 years, 37.9 % between 40 and 55 years, and 34.5 % older than 55 years.

**Data collection** followed two complementary pathways: In order to shed light on the step-by-step development of the workshop, including the obstacles encountered and the facilitating conditions, two researchers systematically documented each stage of the process and discussed the documentation regularly on a weekly basis. This documentation served as a data collection tool, capturing decisions, adaptations, and their underlying rationales throughout the course of the project. In the second pathway, the implementation of the workshops was monitored through observations of the workshops, structured according to a predefined observation protocol with predefined domains. The observation sequences were conducted by three researchers from the project team, with at least two researchers present at each workshop. Prior to the start of every session, the observers briefly introduced themselves and clarified that their role was to monitor the workshop process rather than to evaluate individual participants. Immediately after each workshop, the researchers compared and summarized their key observations and findings, which were subsequently documented for analysis. This also included discussions about who was (in)visible in the room, dominant voices and power dynamics within the group.

Qualitative observation was chosen as the most suitable method for identifying challenges and facilitating factors during implementation and for adapting the workshops to the needs and characteristics of the target group and setting. Previous studies have demonstrated the value of observational approaches in capturing complex, real-world implementation dynamics (73, 74). Furthermore, using observational data instead of self-report minimizes the influence of social desirability bias, which is a common limitation of interview-based methods (75). This strengthens the validity of the findings.

Observational data were stored in a secure institutional folder accessible only to team members.

For **data analysis**, the process documentation and the observation protocols were examined in a structured deductive qualitative content analysis approach (76) to systematically analyze the observation data in relation to the predefined research objectives and research questions. Correspondingly, two researchers developed the deductive coding framework, deriving categories from the research objectives and question. These emphasized the feasibility and practical implementation of the workshops and allowed for a systematic identification of potential barriers and facilitators at both the organizational and conceptual levels. This approach ensured that the analysis remained closely aligned with the objectives of the study while capturing relevant insights across the development and implementation phases. To allow for the inclusion of new and unexpected themes in the data analysis, provided they are relevant to the research, the possibility for inductive coding was also taken into account in the data analysis process.

As a final step, the RE-AIM framework was used to structure the lessons learned from the results and to systematically capture relevant process and outcome dimensions. Key themes were mapped onto its five domains (Reach, Effectiveness, Adoption, Implementation, and Maintenance) and synthesized into practice-oriented implications (see discussion chapter).

The qualitative data analysis software MAXQDA24^1^ was used to manage and analyze the data.

### 2.3 Ethical considerations

The pilot project HCCH was approved by the Ethics Committee of Charité – Universitätsmedizin Berlin (approval number EA1/323/24; approval date: 31 January 2025) and conducted in accordance with the Declaration of Helsinki. All participants have given informed consent.

To ensure the quality of the paper, we have followed the Standards for Reporting Qualitative Research (SRQR).

## 3 Results—tracing the path: insights from the development and implementation process of three WHP workshops

### 3.1 Results I—development process of the workshops

**Figure 1:**
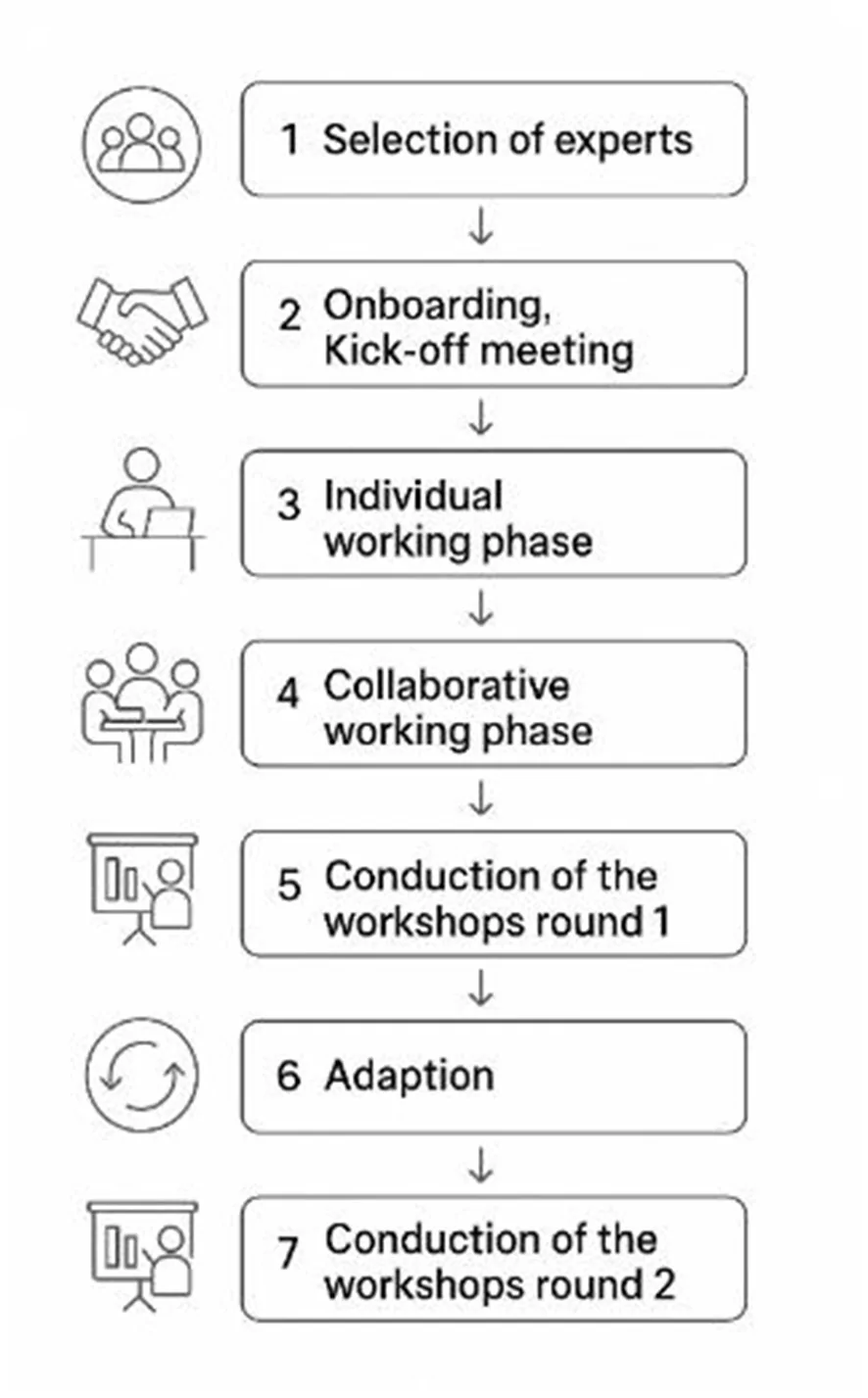
Flowchart development process.

The development and conduction of the workshops took place from September 2024 until July 2025 in a seven-step multi-stage process (see Figure 1)^2^:

**1. Selection of experts**

In the first step, a team of experts was assembled in accordance with the predefined workshop topics (1. nutrition, 2. physical activity, and 3. stress regulation). Relevant selection criteria included expertise in at least one of the workshop topics, experience in conceptualizing and conducting workshops, and interest in, knowledge of, and experience with TCIM. In addition, we sought out experts with experience in setting, target groups, and educational and didactic backgrounds to support the expert group in these areas. For each workshop, a responsible person from the team of experts was appointed to lead the conceptualization and implementation of the workshop: for healthy nutrition (AS^3^), sports and healthy physical exercise (JB^4^), mind-body medicine and stress reduction (AW^5^). They were supported by NE^6^ and SJ^7^. The expert team was led and moderated by two members of the research team: JC^8^ and FV^9^.

**2. Onboarding, kick-off meeting**

In the second step, all experts were introduced to the project in individual face-to-face meetings with the research team. These meetings provided a structured overview of HCCH and outlined the objectives and characteristics of the forthcoming workshops. Building on this shared orientation, the expert group convened for a two-day kick-off, each session lasting 3 h. The meetings served to clarify principles and thematic priorities, address open questions, and generate initial ideas regarding both the content and the structural framework of the workshops.

**3. Individual working phase**

In the third step, the experts engaged in an individual working phase dedicated to the development of the respective workshop. This work was carried out in regular consultation with the research team, ensuring both alignment with the overall project objectives and the integration of emerging ideas into a coherent framework.

**4. Collaborative working phase**

In the fourth step, drafts of the workshop structures and content were first presented and discussed within the expert team, and then with the extended research team. The final adjustments were made based on the feedback received, ensuring that the conceptual design and practical organization of the workshops aligned with the project objectives and that all three workshops had consistent structure and coordinated content.

**5–7. Phase of workshop delivery and final adjustment**

**Step five** involved the conduction of the first round of workshops, in which the experts implemented the previously developed and refined materials. In **step six**, observations and feedback from these sessions were analyzed to identify necessary adjustments, both in content and structure. **Step seven** then comprised the conduction of the second round of workshops, incorporating these adjustments to ensure the workshops were fully optimized for the target group and the setting.

#### 3.1.1 Final workshop structure

Each workshop within the final concept follows a consistent structural design: a workshop session lasts 90 min (see section 3.2.1). After a brief welcome from the facilitator, there is a short introduction to the topic, emphasizing its relevance to the target group. Each workshop covers two subjects (e.g., workshop 1, nutrition: a) healthy eating during shift work; b) putting healthy eating into practice). The theoretical input is complemented by a total of four to six micro-exercises per workshop, which aim to translate theoretical knowledge into experiential learning. For example, in workshop 1 on nutrition, the micro-exercises might include putting together a healthy four-component snack box, reflecting on eating habits at work, mindful eating and developing an individual drinking plan for 1 week. Each workshop concludes with the selection of a *Health Promotion Bundle*, which will subsequently be implemented in the pilot facility during the further intervention process (see Section 1.1.2.).^10^

### 3.2 Results II—process of workshop implementation

The implementation of the workshops involved an ongoing process that took place prior to, during, and following the sessions. Preparations addressing both organizational preparation and the active involvement of staff.

#### 3.2.1 Organizational preparations

A key objective of the pre-implementation phase was to enable as many interested employees as possible to participate in all workshops during their working hours. The intention was challenged by varying work schedules, vacation periods, and sick leaves, as well as employee turnover. It was therefore necessary to engage in regular negotiations and planning discussions with the management of the pilot facility. These discussions took place regularly before and during the implementation phase, primarily through face-to-face meetings, online meetings, and email correspondence.

This led to the following decisions in advance regarding the structure of the workshops and the workshop conditions:

- The duration of the workshops was set at 90 min. The decision was based on discussions with the facility management about the staff's availability, considering shift work and vacation time.
- The workshops were offered as internal training courses and counted as working hours.
- To maximize the participation of the entire staff, irrespective of their work schedules in a shift work system, it was deemed necessary to conduct each workshop twice with about minimum 2 weeks between each session.
- As a result, the six workshops were held over a period of 15 weeks (between March and July 2025).

#### 3.2.2 Staff involvement

During the preparatory phase of the workshops, staff were passively involved through regular updates on the status of the project: the workshops were first announced at a staff meeting in the summer of 2024 after finalization of the needs assessment (24). Another staff meeting in February 2025 was used to inform the staff members about the dates and procedures of the workshops and to generate interest. It was never possible for all staff members to attend team meetings. Thus, a summary was always sent to all employees by email afterwards. Reminder emails were sent approximately 10 days in advance to inform participants of the upcoming workshop dates.

During the workshops, the participants participation was actively engaged through micro-exercises, the proactive moderation of the workshop leaders (e.g., exchange of topics and experiences, integration of participants' contributions into the workshop content, promotion of dialogue), and the joint selection of three *Health Promotion Bundles* (see chapter 1.1.2) for subsequent implementation in the pilot facility.

Following each workshop, participants were provided with a leaflet summarizing key take-home messages and including instructions for the micro-exercises performed. After each workshop had been held twice, the corresponding digital handouts were sent to all staff members, ensuring that non-participants were also informed about the content. The handouts summarized the key topics of the workshops, supported by scientific evidence, and included practical exercises to facilitate implementation in everyday work.

### 3.3 Conducting the workshops: insights and outcomes

All six workshops were observed by at least two and at most three members of the project team. In total, 13 observation reports were compiled. These reports were jointly discussed after each workshop. Any adjustments to the structure and/or content of the workshops arising from these discussions were documented and justified in the ongoing documentation of the implementation process.

#### 3.3.1 Engagement and participants reactions

Each workshop was offered twice to ensure that all members of staff at the pilot facility were able to take part in each workshop once. This organizational decision proved effective in maximizing participation rates. Workshop participation (nutrition: *n* = 20; relaxation: *n* = 26; exercise: *n* = 22) can be considered in relation to a total staff size of *n* = 30–33.^11^ Overall, participation rates ranged from 60.61% to 86.67% (see Table 1). No employee attended the same workshop twice.

**Table 1:** Workshop participation.

Workshop	Round I	Round II	Total number
Workshop 1—Boost your health: eat the natural way	13	7	20
Workshop 2—Boost your health: relax the natural way	17	9	26
Workshop 3—Boost your health: move the natural way	14	8	22

The observations showed that while around two-thirds of participants in most workshops were less involved – one-third expressed interest but remained inactive and another showed no sign of interest or attention – the remaining participants were actively engaged and contributed positively. The positive reactions became apparent in a high level of participation (asking questions, providing answers, engaging in micro-exercises, exchanging content-related views, discussing topics, and contributing their own ideas), in direct verbal feedback on exercises (e.g., ‘very pleasant,' ‘that felt good'), in attentive behavior (nodding, maintaining eye contact, taking notes, smiling in agreement), as well as in enthusiasm and enjoyment (laughter, cheerful atmosphere, active participation, lively discussions about the topics). Moreover, their willingness to engage with the exercises was evident, for instance through concentrated eye-closing during mindfulness practices. Individual participants were also observed sharing ideas for the future with their colleagues, such as trying out micro-exercises with residents, and mentioning that the workshops had provided them with content and inspiration for their everyday practice.

However, a small subset of individuals showed clear signs of dissatisfaction, including verbal objections (e.g., ‘We don't need any more exercise,' ‘Maybe I don't even want to participate'), disengaged behaviors (e.g., distancing themselves from the group, holding private conversations, not paying attention, sighing irritably), and signs of lack of motivation or interest (e.g., no verbal participation, eyes wandering, appearing distracted, slouching tiredly in the chair), suggesting that they perceived the workshops as a waste of time.

#### 3.3.2 Iterative dynamics of facilitators, challenges, and adjustments during workshop implementation

Based on observed reactions and verbal feedback from participants after the first round of workshops, both content-related and structural factors that contributed to the workshops' success, as well as challenges that emerged in their implementation, were identified. These insights served as the basis for maintaining and expanding effective elements while addressing obstacles in the following second round of the workshops.

##### 3.3.2.1 Adequate facilitators for successful workshops

Participants' motivation and interest were supported through didactic elements such as collaboratively developing content, avoiding lengthy monologic theoretical input, and alternating theory with practical micro-exercises for self-experience. The use of a manual flipchart was also positively received, reinforcing the interactive character of the workshops.

In addition, certain behaviors and characteristics of the workshop leaders were found to enhance the workshops' success. This includes:

- Flexible adaptation to participants' interests,
- Clear and effective communication (allowing participants to finish speaking, addressing their topics and questions),
- Linking content to the work setting,
- Encouraging to view even small steps toward health promotion as meaningful achievements,
- Framing content explicitly as recommendations (example: “This is only a recommendation, adaptable to your own needs and possibilities”).

Facilitators also managed restless phases calmly, integrating them into the workshop process while providing clear prompts for quiet when needed.

##### 3.3.2.2 Challenges and barriers for successful workshops

On the other side of the coin, the following areas stood out as challenging: structurally, participants faced a high workload and were often already fatigued from early shifts, which contributed to a noticeable decline in concentration after approximately 30 to 60 min. On a personal level, language barriers among some participants impeded both comprehension and active participation. Content-related factors further affected engagement: some theory input was perceived as excessively long, certain exercises were explained in ways that were too complex to be easily understood by all participants, and specialized terminology in the theoretical segments limited overall accessibility. External disruptions also played a role, including high noise levels from an adjacent kitchen, interruptions from participants on call, and private conversations among attendees.

In addition to content-related and structural factors, group dynamics have also proven to play a significant role in participants' acceptance of the workshops. In particular, resistant attitudes and behaviors displayed by individual participants appeared to strongly influence the overall group dynamic. Workshops in which such resistance was particularly pronounced differed markedly from those in which no participant exhibited a negative stance; in the latter, overall motivation and interest were significantly higher, and engagement with the content was more consistent. In addition, group size proved to be a relevant factor for group dynamic. Sessions with more than 12 participants were characterized by noticeably higher levels of restlessness, whereas a group size of 8 to 12 participants appeared to be optimal.

Dealing with explicit resistance during the workshops proved to be a particularly challenging situation for the workshop leaders. The previously mentioned facilitating characteristics and behaviors of the workshop leaders seemed to positively influence the overall group dynamic and thereby helped, to some extent, to mitigate these challenges. Nevertheless, trust appears to be the key factor in fostering a constructive group dynamic. This includes both the participants' trust in the workshop leaders and the mutual trust established among the group members themselves.

Taken together, these factors highlighted the need for targeted adjustments to both the format and delivery of the workshop to foster sustained attention, comprehension, and motivation.

##### 3.3.2.3 From observations to adjustments

In the second round of the workshops, the facilitating factors were further strengthened. At the same time, content and structures identified as challenging or unsuitable for the target group and/or the setting were adjusted to better align with the participants' needs and the specific conditions of their work context. The overarching aim of the adjustments was to increase the participants' interest and motivation, while reducing their resistant attitudes and behaviors.

Accordingly, multiple aspects of workshop design and delivery were modified: frontal knowledge transfer, which can exacerbate fatigue and reduce motivation, was largely avoided. Instead, short theory blocks of no more than 20 min were implemented, incorporating interactive elements such as guided discussions, joint content development, and quiz games. Each content segment was interrupted at least every 20 min with micro-exercises, allowing participants to actively apply and consolidate new knowledge. The practical exercises in small groups were particularly well received, stimulating valuable discussions and significantly increasing motivation. All exercises were simplified in their description to ensure clarity, and practical examples from participants' everyday work in nursing homes were incorporated to make the content more concrete and relatable. Theoretical input was also streamlined, particularly where specialized terminology was used, and learning objectives were explicitly stated at the beginning of each topic to highlight relevance and prevent resistance.

Workshops were conducted with maximum flexibility and responsiveness to participants' needs and current capacities, fostering trust between leaders and participants. Workshop leaders emphasized approachability, for example by sharing their professional backgrounds and personal connections to the topic, and adapted content on the spot to align with participants' interests, prior knowledge, and experiences. Furthermore, opportunities for discussion were intentionally provided whenever a topic stimulated debate, and the order of micro-exercises was adjusted flexibly to regulate group dynamics and mood.

Collectively, these strategies aimed to enhance engagement, comprehension, and motivation, creating a learning environment that was both supportive and responsive to the challenges identified. The adjustments were evaluated in the second round of the workshops through further observations. The participants' reactions—in particular, positive feedback, active engagement, and visible signs of enjoyment and interest – indicated that these adjustments contributed to greater acceptance and overall success of the workshops.

### 3.4 Lessons learned

The overarching learnings from the process of implementing WHP workshops in a long-term care pilot facility can be grouped into the following subject areas: (1) organizational preparations, and (2) elements of a well-received workshop (a. content, b. structure, c. methods and conditions).

The identified key success factors, summarized in Table 2, provide practical guidance for the future development and implementation of WHP workshops in long-term care facilities.

**Table 2:** Key success factors for the implementation-planning of WHP workshops.

1. Organizational preparations	
**Recommendations for communication during the planning phase**	**AIM**
Communication with facility management (FM) • Long-term planning to enable as many employees as possible to participate, considering ⚬ Shift work planning ⚬ Vacation planning ⚬ Long-term sickness ⚬ Training and further education • Regular communication with facility management via digital meetings, emails, and face-to-face meetings to plan next steps together	= Fostering individual feasibility by cultivating trust, motivation, and perceived involvement through • Planning together (FM) • providing transparent information about the project development (FM + participants) • Reminding about upcoming events (FM + participants) • Opening the door for questions and discussions (FM + participants)
Communication with the participants • Regular communication with the facility staff via email, information sheets and overviews, face-to-face meetings as part of regular employee meetings **Negotiation processes with the facility management regarding**	**AIM**
•Framing the workshops as internal training • Counting workshops as working hours • Availability of resources (space for conducting workshops, usability of facility equipment, e.g., flipchart, pens, notebooks, tables, chairs) • Clarification of logistical issues (e.g., Where can posters/illustrations/information sheets be displayed? Is there a distribution list that can be used to send emails to all participants? How long before and after the workshops can the rooms be used?)	= Fostering organizational and structural preconditions for feasibility
**2. Elements of a good workshop**	
a) Content	•Present content as recommendations to avoid pressure to perform • Use language suitable for the target audience • Avoiding excessive technical terms
b) Structure	•Ideal group size: 8–12 participants • Maximum duration: 90 min to respect participants' resources in a shift-work system • Repeat workshops regularly to allow all staff to attend in a shift-work system
c) Methods and conditions	•Break up theory blocks with micro-exercises • Continuously connect content to participants' work and life context • Allocate time for exchange and discussion • Adjust flexibly to participants' interests • Communicate clearly and effectively, allowing participants to finish their contributions and raise questions • Facilitators should prepare strategies in advance for managing group dynamics, such as handling dominant participants, side discussions, or resistance to activities, such as: ⚬ Set clear rules at the beginning (e.g., respect speaking turns, keep phones silent) ⚬ Communicate the schedule (e.g., breaks, session timings) ⚬ Use active facilitation techniques to maintain staff engagement and keep the workshop flowing (e.g., group discussions, joint collection and structuring of knowledge) ⚬ Address disruptions calmly and directly ⚬ Redirect off-topic discussions by summarizing and linking back to the workshop's goals ⚬ Encourage balanced participation, ensuring that quieter participants also have a voice ⚬ Maintain a supportive tone, reinforcing constructive behavior rather than focusing on mistakes

## 4 Discussion—from observation to insight: lessons learned in developing and implementing WHP workshops

The goal of this paper was to outline the development process of the *HCCH* workshops, to make the underlying decisions and considerations traceable. Furthermore, drawing on the facilitators and pitfalls encountered during implementation, the paper sought to develop guiding implications to inform the planning and execution of similar interventions in comparable contexts.

Over the course of the seven-phase process from October 2024 to July 2025, the three *HCCH* workshops were conceptualized and implemented. Through early and consistent planning with the management of the pilot facility and by framing the workshops as part of internal professional training, a notably high participation rate was achieved (number of participants ranged between min. 60.61% and max. 86.67%). Thus, one of the key factors to the successful implementation of WHP programs—the alignment of WHP activities with shift schedules (15)—was already addressed during the planning phase.

Facilitators and challenges in regard to the workshop implementation were identified based on both positive reactions and signs of dissatisfaction among participants, as captured through structured observation sequences during the first round of the workshops. These insights highlighted the need for adjustments to the workshops' structure and delivery to sustain attention, enhance comprehension, and increase motivation. Participants' engagement and positive responses during the second round of workshops suggested that these modifications effectively supported acceptance and overall success.

Building on these multifaceted findings, it was possible to derive implications for the successful implementation of workplace health promotion workshops—structured by the RE-AIM model –, which will be outlined and discussed in the following chapter (see Table 3).

**Table 3:** Interpretation of findings and practical implications structured by the RE-AIM framework.

RE-AIM domain	Interpretation of findings	Implications for practice
Reach	•High participation indicates that WHP interventions are feasible in long-term care when organizational conditions and communication strategies are aligned with staff needs • Structural factors such as shift work and information access remain critical determinants	•Align workshop scheduling with shift systems and staff availability • Use coordinated, transparent, and repeated communication strategies across multiple channels to ensure broad awareness and accessibility
Effectiveness	•Evidence on effectiveness remains limited due to the qualitative process evaluation design • However, expressed intentions to apply learned strategies suggest perceived relevance and potential behavioral impact	•Focus on practical relevance by linking content closely to everyday work and life contexts • Use accessible language and emphasize actionable strategies to support transfer into practice
Adoption	•Adoption is shaped by both organizational readiness and individual engagement • Management support and integration into routine structures are key facilitators • Lack of interest may limit uptake	•Embed WHP interventions within organizational routines (e.g., recognition as working time, provision of infrastructure) • Involve management early and promote shared ownership (= active participation) to enhance uptake
Implementation	•Implementation was the central domain of the study • Relevant aspects included engagement, group dynamics, and contextual constraints (e.g., time pressure, fatigue, language barriers) strongly influencing delivery • Iterative adaptation proved essential for feasibility and acceptability	•Design flexible, resource-sensitive workshop formats (short duration, repeated sessions, small groups) • Apply interactive and participatory methods • Equip facilitators with strategies to manage group dynamics and adapt to participants' needs
Maintenance	•Due to the feasibility-oriented, single-arm observational design of this study, no statements can be made about the long-term effects on either the individual or organizational levels. This aspect will be addressed in the subsequent implementation phase (start in September 2026) and its evaluation • Sustainability is likely to depend on continued organizational support, although this could not be evaluated within the scope of the present study	•Promote sustainability through integration into existing structures (e.g., regular meetings, ongoing training formats) • Maintain continuous communication and consider follow-up activities to reinforce long-term impact

### 4.1 Lessons learned for WHP workshops' implementation in longterm-care facilities—a RE-AIM perspective

This chapter synthesizes the key lessons learned from the implementation of WHP workshops in long-term care facilities through the lens of the RE-AIM framework (Reach, Effectiveness, Adoption, Implementation, and Maintenance) (77, 78). RE-AIM is used here as an organizing heuristic to structure the interpretation of the qualitative results and to facilitate a systematic reflection on both process and outcome dimensions of the intervention. Using RE-AIM as an analytical framework offers several advantages for summarizing complex implementation experiences. It enables a multidimensional assessment that goes beyond efficacy alone by explicitly considering the extent to which the intervention reached the target population, the degree to which it was adopted by settings and staff, the fidelity and variability of implementation, and the potential for sustained use over time. This helps to disentangle context-specific barriers and facilitators at each stage of the implementation pathway. Moreover, structuring the findings according to RE-AIM provides a transparent and replicable way of reporting that highlights not only whether the intervention worked, but under which conditions, for whom, and with what level of feasibility in real-world care settings. In doing so, the framework contributes to translating empirical insights into actionable guidance for future implementation efforts in long-term care environments.

### 4.2 Target group participation in WHP projects

Most of the success factors summarized in Table 2 are intended to foster interest, motivation, and identification of the target group with the WHP program. These elements are considered important for the WHP long-term implementation and success, as sustainable implementation is more likely when employees identify with and are intrinsically motivated to engage in such programs (79). To generate and maintain motivation and identification over time, active involvement and participation of the target group are regarded as crucial facilitators (79, 80). However, despite the widespread recognition of workplace WHP programs as effective means to enhance employees' health and wellbeing, achieving consistent and long-term participation remains a well-documented challenge (80). Moreover, the actual willingness of healthcare employees to participate in WHP programs is often overestimated [7: 50ff]. In fact, WHP programs are at times even perceived as an additional burden by the target group, which can further reduce motivation to engage in such interventions (16, 17). This highlights a gap between the theoretical acknowledgment of WHP's relevance and the practical readiness for engagement. The discrepancy was also observed during the workshops, where indications of limited engagement and some resistance among certain participants pointed to varying degrees of participation readiness within the group (see chapter 3.3.2.2.).

The following themes have been discussed as possible explanations: existing evidence suggests that employees' hesitation to participate in WHP initiatives may stem from several underlying factors. In some cases, this reluctance reflects a general lack of interest; in others, it is driven by concerns that the employer might either fail to implement meaningful changes or intervene inappropriately, as well as by a broader fear of change [7: 49]. Moreover, limited openness and a lack of problem awareness can further impede participation:

Developing and maintaining motivation among the target group is also considered a key success factor for successful behavioral change in WHP projects: according to the Transtheoretical Model of Behavior Change (TTM), initially developed by Prochaska and DiClemente (81–84), the degree of individual motivation plays a decisive role in whether behavioral change is initiated and maintained. The TTM conceptualizes behavioral change as a dynamic process that unfolds through a series of distinct stages, rather than as a single event. These stages are: precontemplation (no current intention to change); contemplation (awareness of the need for change and consideration of its benefits); preparation (intention to act in the near future); action (active implementation of new behaviors); and maintenance (sustained behavior and prevention of relapse) (81–84). To successfully and sustainably implement workplace health promotion (WHP) initiatives, it is therefore essential to consider these stages of change and tailor interventions to the respective level. Motivation and awareness are, according to TTM, key during the stages of contemplation and preparation, which represents optimal phases for initiating behavioral change. By contrast, the stage of precontemplation—characterized by low awareness and absent motivation—constitutes a far more challenging starting point for engagement (85). Targeting these stages can therefore enhance the effectiveness of WHP initiatives by aligning intervention strategies with employees' motivational readiness for change. When the target group shows heterogeneous levels of motivation, with some participants possibly still in the precontemplation stage, communicating and implementing low-threshold, accessible interventions that can be easily integrated into daily routines can be a valuable first step [see e.g., (16, 86)].

Another explanatory perspective on the resistance observed toward certain WHP activities, such as mindfulness exercises, relates to the psychological strain and exhaustion often experienced by healthcare professionals [e.g., (3)]. For employees already operating under high workload and chronic stress, practices that require self-reflection or heightened bodily awareness may unintentionally evoke discomfort rather than relief. Resistance in this context might therefore be understood less as a lack of interest or openness, and more as a protective mechanism [see e.g., (87)]. Confronting one's physical and emotional state might make the extent of exhaustion and overload consciously perceptible—an experience that, for some, might feel threatening or incompatible with the need to maintain functionality in daily work. Consequently, avoidance or resistance may serve as a coping strategy to preserve the ability to continue working despite persistent strain. It is also indicated that time constraints, feelings of guilt for prioritizing self-care over patient care, and fear of vulnerability in front of others may hinder participation in interventions involving mindfulness strategies (88).

All explanatory approaches addressing the low willingness to participate in WHP programs highlight an inherent paradox: participation is both the prerequisite for and the outcome of developing motivation and sustained engagement.

Given the high relevance of this topic for the successful implementation of WHP programs, the active participation of the target group was considered from the very beginning in the development and implementation of the *HCCH* workshops. Starting with a needs assessment to identify health-related needs, demands, and conditions from the perspective of the target group (24), further involvement included regular in-person information sessions, emails, handouts, and notices. The aim of communication via these channels was to provide transparent information about further developments, give an overview of planning steps, conduct surveys, and stimulate discussion in order to encourage a sense of self-efficacy within the target group. This form of involvement can be classified as a preliminary stage of participation (29). Yet, the implementation of the workshops allowed for full participation through the selection of *Health Promotion Bundles (HPB)* by the participants. According to the staged model of participation by Wright et al. (29), this corresponds to the levels of consultation and shared decision-making. Participation will be progressively expanded throughout the further project process in order to support the sustainable implementation of the WHP program.

Future research and practice within the field of WHP projects may benefit from examining how a higher level of participation can be fostered from the outset without overburdening the target group's resources.

Motivating and engaging participants who initially demonstrate disinterest or resistance in workplace health promotion (WHP) programs requires careful consideration of trust and relational dynamics. Trust has been widely recognized as a crucial factor in organizational contexts, influencing individuals' willingness to engage in new initiatives and to commit to sustained participation (7). Within the context of WHP programs, establishing trusting relationships between participants and program facilitators or organizational leadership can therefore be decisive.

Empirical findings suggest that combining meaningful participatory elements with a positive, trust-based relationship to management strengthens participants' commitment to WHP initiatives (79, 89). When employees perceive that their input is valued and that program leaders are approachable and supportive, initial resistance can be mitigated, and engagement increased. Conversely, negative associations with the employer can function as significant barriers, reducing willingness to participate and undermining program outcomes (15).

Future research should take up the question of how trust can be deliberately fostered as part of WHP interventions, in order to further illuminate the multifaceted potential of relational dynamics as a key facilitator of successful WHP programs.

### 4.3 Strengths and limitations

The use of accompanying qualitative observations during the workshops proved to be a suitable method for identifying both, challenges and facilitating factors in the implementation process. Based on the insights gained from these observations, the workshops could be refined and adapted in accordance with the specific needs of the target group and the contextual characteristics of the respective settings. Observational approaches have been shown to capture complex real-world implementation dynamics effectively and can also complement RCTs, helping to reveal contextual factors behind outcome-focused quantitative measures and guiding necessary adjustments to interventions (73, 74). By relying on observational sequences rather than self-reported data, the influence of social desirability bias—commonly observed in interview-based methods (75)—was effectively minimized. This strengthened the validity of the data and provided a more authentic understanding of participants' interactions and engagement within the workshops.

A key strength of this study lies in the research team's diverse and well-founded expertise in qualitative research. Continuous exchange regarding methodological decisions and procedures ensured a thorough and critical reflection on the applied approaches. This collaborative process enhanced the study's methodological rigor and credibility, allowing for well-grounded interpretations of the observational data. Furthermore, the involvement of multiple researchers in both, data collection and analysis contributed to investigator triangulation and helped to minimize individual bias. In particular, this concerns a potential role conflict involving two researchers (JC, FV), who were part of the observer team (consisting of three team members in total) as well as involved in co-developing the workshops. This overlap in roles may have introduced potential biases, such as expectation effects or reduced critical distance toward the intervention's outcomes. To mitigate this potential bias, each observation was independently documented by the team members. The observations were then compared and discussed to identify potential discrepancies in interpretation (see chapter 2.2.1, “data collection”). Additionally, observations were regularly shared and discussed with members of the extended research team (WS, GS, CK). A temporary irritation of the workshop dynamics due to the observation's setup was noted only at the beginning of the first two sessions, when participants were still unfamiliar with the situation. Such initial tension was promptly addressed by introducing the observers and clarifying that the observations served the purpose of improving the workshops rather than evaluating individual participants' behaviors. As the implementation progressed, the presence of observers no longer affected the group dynamics. However, the data collection method was limited to observational field notes and accompanying process documentation. Consequently, the data cannot provide evidence regarding the effectiveness of the intervention itself. While such evaluation was not the objective of this feasibility-focused study, the aspect of the intervention's effectiveness is being addressed in a currently ongoing mixed-methods evaluation accompanying the intervention's broader implementation.

Beyond the limitations related to the data source itself, the analytical perspective adopted in this study should also be considered: given that several implementation challenges concerned participants' engagement, motivation, and perceived relevance of the workshop content, the role of an explicit adult learning perspective should also be considered. Adult learning theories were not incorporated into the initial workshop development or the analytical framework. Drawing on theories such as Knowles' theory of andragogy (90) or Kolb's experiential learning theory (91), could inform the design and evaluation of future WHP workshops.

A further limitation concerns the interpretation of higher levels of acceptance of the adjusted workshops observed among participants in the second round. This kind of improvement cannot be attributed unambiguously to the workshop modifications alone, as participant numbers were consistently lower in the second round compared to the first. In fact, the reduction in group size may also have contributed to the improved acceptance. Such an alternative interpretation would lend additional support to our recommendation of a group size of 8–12 participants per workshop (see Table 2, “*Elements of a good workshop”*).

For completeness, it should be mentioned that the workshops involved only a single participant group (staff of the pilot facility) and one setting, which may limit generalizability to other contexts in which different challenges could arise. Additionally, the effectiveness of the workshops depends on the facilitators' engagement, skills, and style, which can influence outcomes. These contextual factors should be considered in future WHP initiatives and evaluations and are further addressed in the following prospects.

### 4.4 Prospective workshops

All three workshops will be repeated by new facilitators to examine the extent to which they can be successfully implemented using only the guidelines and materials provided. This procedure does not only serve to validate the feasibility of the workshops, but also to assess the transferability of the materials to different settings. Where necessary, the guidelines will be refined based on these insights. Notably, the assessment of Workshop I—“*Boost Your Health: Eat the Natural Way”*—was already successfully completed in July 2025 with a target group of medical students and a new workshop facilitator (a psychologist and health scientist, WS). In the medium term, the transfer of the workshops to other long-term care facilities as well as their evaluation is planned. For this purpose, a comprehensive manual including instructions and contextual information will be developed.

## 5 Conclusion

The objective of this paper was to outline the development process of three WHP workshops on nutrition, stress regulation and physical activity, and to make the underlying decisions and considerations transparent. A second objective was to derive practical recommendations for planning and implementing similar interventions in comparable contexts by considering the facilitators and challenges involved.

These objectives were identified based on signs of agreement, interest, and positive reception, as well as on indications of dissatisfaction, captured through qualitative observation sequences among participants during the first round of workshops. Insights from these observations informed adjustments to the structure and delivery of the workshops, improving participants' attention, comprehension and motivation. Participants' engagement and positive responses in the second round indicated that the modifications had effectively enhanced acceptance and the overall success of the workshops.

Key learnings from this process could be grouped into two categories: (1) organizational preparations and (2) elements of a well-received workshop, covering content, structure, and methods/conditions. Notably, the active participation of the target group was identified as a critical factor in fostering motivation and a sense of ownership. Participation should therefore be considered both, a prerequisite for successful WHP projects and a key outcome of their effective implementation. This highlights the urgent necessity of involving participants in the development of workplace health promotion interventions. The synthesis and interpretation of these key learnings in accordance the RE-AIM framework underscores the practical relevance of the findings by integrating reach, effectiveness, adoption, implementation, and maintenance into a transparent interpretation that facilitates translation into real-world practice.

## Data Availability

The datasets presented in this article are not readily available because the dataset contains qualitative data with potentially identifiable information. Public sharing of the raw data is restricted due to participant confidentiality and data protection requirements. Access may be granted in exceptional cases upon reasonable request and subject to ethical approval and applicable legal regulations. Requests to access the datasets should be directed to judith.czakert@charite.de.

## Footnotes

1 VERBI Software. MAXQDA 2025. Berlin: VERBI Software; 2025.

2 Graphic by ChatGPT. August 18^th^, 2025. Prompt: *Create a flowchart for me to illustrate the following seven steps (which took place chronologically) for publication in a scientific journal: 1. Selection of experts 2. Onboarding, Kick-off meeting 3. Individual working phase 4. Collaborative working phase 5. Conduction of the workshops round 1 6. Adaption 7. Conduction of the workshops round 2*.

3 Nutritional Scientist and Master of Public Health (MPH).

4 Applied Exercise Scientist, Therapeutic Climbing Instructor.

5 Psychologist, Mind-Body Medicine Therapist, Aromatherapist.

6 Health Educator, Trainer and Lecturer in Behavior Change, Communication, Stress Management, Relaxation Techniques.

7 Teacher in Movement and Social Sciences, Registered Nurse, Health Coach.

8 Registered Nurse, Nursing and Health Scientist, Master's degree in Cultural Anthropology (M.A.).

9 Registered Nurse and Nursing Scientist.

10 The final workshop materials were originally developed in German and can be made available upon request. A translation into English is currently not available.

11 Due to staff turnover during the implementation period, the number of employees decreased from 33 to 30.
